# Effectiveness of self‐management interventions on Type 2 diabetes among young adults (18–45 years): A systematic review and meta‐analysis

**DOI:** 10.1111/dme.70127

**Published:** 2025-08-21

**Authors:** Sonia Khavere, Michelle Hadjiconstantinou, Joanne Miksza, Jenny Hagan, Shukrat Salisu‐Olatunji, Sara Naderpour, Sarah Nalir Hassen, Zahra Karimi, Clare L. Gillies

**Affiliations:** ^1^ Department of Population Health Sciences University of Leicester Leicester UK; ^2^ Diabetes Research Centre, College of Life Sciences University of Leicester Leicester UK; ^3^ NIHR Leicester Biomedical Research Centre University of Leicester, Leicester General Hospital Leicester UK; ^4^ Leicester Real World Evidence Unit, Leicester Diabetes Centre Leicester UK

**Keywords:** diabetes mellitus, Type 2, meta‐analysis, self care, self‐management, systematic review, young adult

## Abstract

**Aims:**

Self‐management interventions effectively improve health outcomes in adults with Type 2 diabetes. Young adults living with Type 2 diabetes are at a higher risk of diabetes‐related complications, hospitalisation and reduced quality of life. While self‐management is key in diabetes care, its effectiveness in young adults with Type 2 diabetes remains unclear. This review assessed self‐management interventions' impact on health outcomes in young adults (18–45 years) with Type 2 diabetes.

**Methods:**

Five electronic databases were searched from inception to May 2024. Trials evaluating self‐management interventions versus usual care in young adults were included. Outcomes of interest were clinical, self‐care behaviour and psychological health. Meta‐analysis used a random effects model; study quality was assessed using the Cochrane risk of bias tool (RoB2) and the JBI checklist for quasi‐experimental studies. The review followed PRISMA guidelines and was registered on PROSPERO (CRD42024522979).

**Results:**

Ten studies were included, nine in the meta‐analysis, which reported no significant differences between the intervention and control groups in HbA1c, body mass index, weight, waist circumference, blood pressure, lipids, depression or self‐efficacy outcomes. Nevertheless, these findings are imprecise due to few studies, missing data and small sample sizes. Commonly used behaviour change techniques were goals and planning, shaping knowledge and feedback/monitoring.

**Conclusions:**

Existing self‐management interventions did not improve clinical and psychological outcomes among young adults (18–45 years) living with Type 2 diabetes. More effective strategies are needed for this priority population.

**What's new?**

**What is already known?**

Type 2 diabetes (T2D) has substantially increased among adults under 45 years who show poorer outcomes compared to those >45 years. Though self‐management interventions (SMIs) are effective in T2D management among adults >18 years, no review has assessed SMIs' effectiveness in 18 to 45‐year‐olds.

**What this study has found?**

Current SMIs did not improve blood glucose control, weight, cardiovascular health indicators, depression levels or self‐efficacy of adults under 45 years living with T2D.

**What are the implications of the study?**

There is a critical need for effective self‐management strategies for younger adults under 45 years living with T2D.

## 1 INTRODUCTION

Worldwide, about 15%–20% of individuals living with Type 2 diabetes (T2D) are young adults.^1^ The prevalence of T2D among young adults 20–39 years has quadrupled between 2013 and 2021,^2^ with a similar upward trend in incidence rates. The incidence rates of T2D among individuals 15–39 years have more than doubled, 56.4%, between 1990 and 2019.^3^ Early onset of T2D at <40 years is linked to two to three times faster deterioration in beta function, higher probability of hospitalisation,^4^ diabetes‐related complications,^4^, ^5^ reduced quality of life and life expectancy^4^ compared to later onset at >40–45 years. Notably, the disability‐adjusted life years among this group have risen by 40% between 1990 and 2019.^3^

The chronic nature of T2D requires the individual living with the condition to actively participate daily in self‐care activities to control hyperglycaemia and prevent diabetes‐related complications. This type of management by individuals living with the condition is termed self‐management, which comprises the majority of diabetes management.^6^ The American Association of Diabetes Educators breaks down self‐management into seven self‐care activities, including self‐compassion, medication adherence, a healthy diet, glucose monitoring, decreasing the probability of complications, being physically active and problem‐solving.^7^ Self‐management is mainstay in diabetes management,^8^ and self‐management interventions (SMIs) provide support and increase confidence in participation in self‐care for overall health and well‐being.^9^

SMIs among adults >18 years have been effective in improving glycaemic control; nevertheless, mixed evidence has been reported on other health outcomes, with recent findings in favour of SMIs. A meta‐review of SMI studies of mixed study design published between 2001 and 2016 among adults over 18 years reported a clinically significant improvement of −3 mmol/mol (−0.25%) and −5 mmol/mol (−0.5%) in glycated haemoglobin (HbA1c) and inconsistent improvements in other health outcomes.^10^ However, an updated review of more recent randomised controlled trials (RCTs), 2000–2018, found statistically significant improvements in HbA1c and other clinical, behavioural, and psychological outcomes apart from high‐density cholesterol and psychological distress^9^ among adults >18 years. The high‐quality evidence from the latter review underscores the effectiveness of SMIs in managing T2D and enhanced health and well‐being.

Despite evidence supporting the effectiveness of SMIs in RCTs involving adults over 18, it is important to highlight that young adults living with T2D are often underrepresented in clinical trials. A review of 90 RCTs that have informed the development of diabetes management guidelines found that only 73 of these trials included young adults, 18–39 years, who made up less than 5% of the participants.^1^ This is particularly concerning given that young adults constitute 15%–20% of the total adult population living with T2D.^1^ This underrepresentation suggests that current diabetes management guidelines may be biased towards the needs of older adults with T2D, potentially neglecting the unique challenges and requirements faced by younger populations. Young adults living with T2D often deal with unique challenges such as stigma, time constraints from balancing self‐management with work, education or family responsibilities,^11^ and have poorer health outcomes and metabolic risk profiles compared to older adults.^4^, ^5^ Therefore, evaluating the effectiveness of current SMIs targeting young adults living with T2D is critical in informing the development of effective diabetes management guidelines and interventions for this group.

To date, only one review^8^ has been conducted to evaluate the effectiveness of self‐management interventions among young adults. However, the review focused on those living with Type 1 and Type 2 diabetes. At the time, few of the included studies, two out of 13, specifically targeted young adults with T2D, which limited the evaluation of the effectiveness of SMIs among this target group. Recently, evidence on SMIs among young adults with T2D has increased; this review, therefore, aimed at estimating the effectiveness of SMIs on clinical, behavioural and psychological outcomes of young adults, 18–45 years, living with T2D. Additionally, behaviour change techniques were synthesised to identify the active components of the interventions that contributed to behaviour change.

## 2 REVIEW METHODS

This review followed the Preferred Reporting Items for Systematic Reviews (PRISMA) (2020) reporting guidelines.^12^ The review protocol was registered on PROSPERO on 25 March 2024, registration number CRD42024522979.

### 2.1 Search strategy and study selection

The Medline, Cochrane, CINAHL, Web of Science and Scopus databases were searched from inception to 29 May 2024. The search strategy was developed using Medical Subject Headings (MeSH) and free‐text keywords, first on Medline and then tailored for the other databases (Table S1). Terms used were related to Type 2 diabetes, self‐management and randomised controlled trials (RCTs). Due to limited resources for translation, databases were limited to articles published in English.

The key inclusion criteria were studies whose participants were young adults, 18–45 years, living with Type 2 diabetes as a subgroup or whole population or whose trial datasets were available to extract the outcomes of the population of interest. We included trials evaluating the effectiveness of self‐management interventions in young adults compared to standard, usual care or no intervention. All self‐management interventions targeted to improve one or more of the seven self‐care activities described by the American Association of Diabetes Educators^7^ were included. The primary outcome of interest was glycated haemoglobin (HbA1c), and secondary outcomes were clinical, psychological and behavioural outcomes, such as body mass index, blood pressure, diabetes distress, self‐efficacy, physical activity or diet‐related.

### 2.2 Screening, data extraction and quality assessment

The search results were combined in Endnote where duplicates were removed. The remaining retrieved articles were uploaded to Rayyan for screening. Five blinded reviewers (SK, SN, SNH, ZK and CG) conducted the initial title and abstract screening, while four blinded reviewers (SK, JH, JM and SSO) conducted the full‐text screening. Conflict resolution was done through consensus among the reviewers.

Data were extracted independently by two reviewers (SK and JM) using a tailored data extraction tool in Excel, which was adapted from Schmidt et al. (2020).^13^ Outcome data of young adults (18–45 years) from published trial datasets were also extracted. In addition to outcome data, the study and intervention characteristics, main findings, study strengths and limitations, funders and conflict of interest data were extracted. In two studies^14^, ^15^ where more than one intervention was employed compared to control, outcome data were extracted for the most intensive intervention; in one case, outcome data of participants in a thrice‐weekly structured exercise intensive group were extracted over the once‐weekly intensive. Additionally, the outcome data of participants in a motivational interviewing intensive group were selected over those in the self‐management education intensive group; the motivational interviewing intervention included an education component and additional support sessions with a psychologist and nutrition expert. Cross‐referencing of the extracted data for accuracy was conducted, and disagreements were resolved through consensus. Contacting study authors for missing data or values was not conducted.

The Cochrane risk of bias tool (RoB2 checklist) and the JBI checklist for quasi‐experimental studies were used to assess the risk of bias. The RoB2 tool evaluated possible bias based on five domains: randomisation process, deviations in implementation of intended interventions, outcome data missingness, outcome measurement and reporting of study results.^16^ Contrastingly, the JBI checklist for quasi‐experimental studies evaluated bias related to temporal precedence selection and allocation, confounding factors, intervention administration, outcome assessment, detection and measurement, participant retention and the validity of the statistical conclusion. Two reviewers (SK and CG) independently conducted the quality assessment and provided individual judgments for each domain. The judgments were classified as low risk, high risk or some concerns according to the guidance in the Revised RoB2 guidance document for parallel trials by Higgins et al.^17^ and the JBI checklist for quasi‐experimental studies.^18^ Discrepancies among the reviewers at the domain level were resolved through consensus, and the reviewers agreed upon the overall judgment. RCTs were categorised as low risk if four or more domains were overall classified as low risk, high risk if four or more domains overall were classified as high risk and the remaining studies that did not meet the above criteria were given the overall judgment as some concerns. Quasi‐experimental studies were categorised as low risk (high quality), some concerns (moderate quality) or high risk (low quality) if 7–9, 4–6 and 1–3 of the questions were marked yes, respectively.

### 2.3 Data synthesis and analysis

The data from young adults included within the four open‐access datasets^14^, ^19^, ^20^, ^21^ were analysed to compute mean values, standard deviations (SD), mean differences (MD) and standard error (SE) for outcomes of interest. A complete case analysis was performed. The means were reported unadjusted in accordance with the outcome data of the other included studies. Additional calculations were also carried out for some of the included studies where individual participants' data was not open access. This included calculating the MD (change from baseline in intervention group – change in baseline from control group) and SEs, where these were not reported, using baseline and follow‐up data. For two such studies,^15^, ^22^ the sample sizes of the control and intervention groups were not explicit, and limited calculation of the SEs was conducted. Clarification was sought from study authors, and following a lack of response, the sample in each was assumed to be split equally across all study arms.

Pooled mean differences with their corresponding 95% confidence intervals (CI) and *p*‐value were computed using the random effects meta‐analysis model. Standardised mean differences were computed for depression and self‐efficacy variables, which were reported using different units across studies. For all other outcomes, mean differences were used as units were consistent across studies. The random effects model was deemed appropriate due to variations between studies in methodological quality and types of self‐management interventions employed. Heterogeneity was quantified using the *I* ^2^ statistic,^23^ and an *I* ^2^ of 75% or greater was considered high heterogeneity.^24^

Subgroup analysis was performed for the outcome of HbA1c as this was reported most consistently across studies, to evaluate the effect of study quality (some concerns vs. low risk) and mode of delivery of the intervention (digital vs. non‐digital). Assessing the effectiveness of digital interventions was critical as they have been proposed as a promising approach to support young adults living with T2D, who often receive less clinical monitoring than those with Type 1 diabetes.^25^ Furthermore, traditional face‐to‐face support services may not be readily accessible or scalable to meet the growing needs of this group.^25^ Publication bias was evaluated visually using funnel plots and statistically using Egger's regression test. The data were analysed using StataCorp. *Stata Statistical Software: Release 18*. College Station, TX: StataCorp LLC; 2023.

Study and intervention characteristics, behaviour change theories, and behaviour change techniques utilised in the interventions were narratively synthesised and presented in tables. Also, outcome data that could not be pooled in the meta‐analysis were narratively synthesised.

## 3 REVIEW RESULTS

**FIGURE 1:**
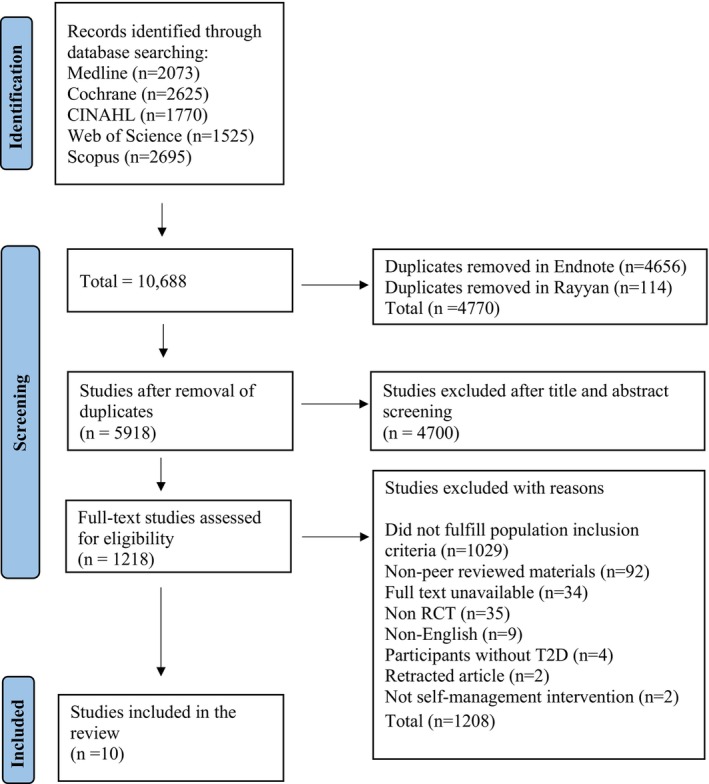
PRISMA flow diagram.

A total of 10,688 articles were retrieved from the database search, and after de‐duplication, 5918 titles and abstracts were screened. The remaining 1218 articles underwent full‐text screening, and 10 articles were included in this review; 9 were included in the meta‐analysis. The PRISMA flow diagram presents the reasons for exclusion in the full‐text screening (Figure 1).

The majority of the studies were conducted across the United States of America (USA) (*n* = 4)^20^, ^21^, ^26^; the remaining (*n* = 6) were conducted in Iran (*n* = 2),^15^, ^22^ Australia (*n* = 2),^27^, ^28^ South Korea (*n* = 1),^29^ and Nigeria (*n* = 1).^19^ Concerning intervention delivery, SMIs were delivered through the hospital (*n* = 1),^19^ digitally (*n* = 3),^15^, ^28^, ^29^ community centre (*n* = 1),^14^ or in combinations that included community centres and participants' homes (*n* = 1),^21^ community centre, homes and digital (*n* = 1),^26^ homes and digital (*n* = 1)^20^ and participant's home (*n* = 1)^27^; one study did not report the delivery of its intervention.^22^ All included studies were trials; eight were randomised control trials, while two^15^, ^22^ were quasi‐experimental.

Five studies^15^, ^22^, ^26^, ^27^, ^28^ were explicitly employed among young adults; participants in four of these studies were living with T2D,^15^, ^22^, ^27^, ^28^ while one included those living with T1D and T2D.^26^ The remaining five studies included all adults (18+ years) living with T2D; one of these studies^29^ reported a sub‐group analysis of those under 45 years, while the remaining four^14^, ^19^, ^20^, ^21^ had open access trial data published where data of the target population was extracted. In the latter, the age range of the young adults was 24–45 years. The sample sizes of young adults aged 18–45 in the included studies ranged between 20 and 227. On average, participants had lived with diabetes for between 1.00 ± 0.00 and 8.10 ± 6.61 years. Table 1 reports a summary of the study and participant characteristics.

**TABLE 1:** Study and participants characteristics.

Author, year	Country	Setting	Sample size	Age range	Women (%)	T2D duration (years)
Abdollahi et al., 2020^22^	Iran	Unclear	30	35–45	N/R	≥1
Dibaiyan et al., 2022^15^	Iran	Digital	45	25–45	66.6	≥1
Cho et al., 2017^29^	South Korea	Digital	Total: 484 <40 years: 34	N/R	N/R	≥1
Gerber et al., 2023^20^	USA	Digital +home visits	Total: 221 Extracted: 30	Total: 21–75 Extracted: 27–45	Total: 69.7 Extracted: 66.7	Total: 12.7 (7.8)^Ø^ Extracted: 8.1 (6.61)^Ø^
Lake, 2020^27^	Australia	Homes	227	19–39	56	1.6 (2.5)^Ø^
McElfish et al., 2021^21^	USA	Community centre+ homes	Total: 221 Extracted: 35	Total: N/R Extracted: 32–45	Total: 56.8 Extracted: 48.6	Total: N/R Extracted: N/R
Mukherji et al., 2022^14^	USA	Community centre	Total: 357 Extracted: 55	Total: 18–80 Extracted: 27–45	Total: 40.1 Extracted: 40.0	Total: N/R Extracted: N/R
Middleton et al., 2021^28^	Australia	Digital	40	18–40	50.0	6.4^Ø Ø^
Essien et al., 2017^19^	Nigeria	Hospital	Total: 118 Extracted: 20	Total: >18 years Extracted: 24–45	Total: 60.2 Extracted: 70.0	Total: 6.4 (4.3)^Ø^ Extracted: 4.25 (3.2) ^Ø^
Pyatak et al., 2018^26^	USA	Homes + community centre + digital	Total: 81 T2D: 20	18–30	Total: 63.0 T2D: N/R	≥1

*Note*: ^Ø^, mean (SD); ^Ø Ø^, mean; total: characteristic of the whole included sample of adults >18 years; extracted: characteristic of included sample 18–45 years.

Abbreviation: N/R, not reported.

### 3.1 Characteristics of self‐management interventions

The type of self‐management interventions employed varied significantly between the studies and included acceptance and commitment therapy,^22^ motivational interviewing,^15^ an internet‐based health gateway device,^29^ mobile health,^20^ a health promotion leaflet,^27^ occupational therapy,^26^ an enhanced SMS‐based support and reminder programme,^28^ structured exercise,^14^ and diabetes self‐management education.^19^, ^21^ Most studies' treatment and follow‐up durations ranged between 8 weeks and 6 months, and 1 and 24 months, respectively.

Six studies reported tailoring their interventions. Of these, one study^27^ tailored its intervention for young adults aged 18–39 years, while four studies^20^, ^26^, ^28^, ^29^ tailored their interventions to individual participants based on various factors, including personal goals, preferences, needs, baseline characteristics, or smoking status. One study^21^ tailored its intervention to fit a particular culture but did not specifically tailor the intervention for young adults. In three studies,^14^, ^15^, ^22^ it was unclear whether any tailoring had been done. One study^19^ explicitly stated that no tailoring was planned. Out of the 10 studies, three were group‐based,^14^, ^15^, ^19^ four^20^, ^27^, ^28^, ^29^ were individual‐based, two^21^, ^26^ allowed participation from both individuals and their family members, and in one study,^22^ it was not specified to whom the intervention was delivered.

Two studies^26^, ^29^ recorded adverse events; however, after assessment, they were deemed to be unrelated to the study procedures. Two other studies^20^, ^27^ assessed adverse events but found none, while the remaining studies (*n* = 6) did not report on the assessment of adverse events. Full details of the interventions are presented in Table 2.

**TABLE 2:** Intervention characteristics.

Author	Intervention type	Study duration	Follow‐up	Frequency, timing and duration of intervention	Tailored intervention	Individual or group‐based	Delivery team	Adverse events	Control type
Abdollahi et al.^22^	Acceptance and commitment therapy	6 months	6 months	8 sessions of 45–60 min once per week	Unclear	Unclear	N/R	N/R	No intervention
Dibaiyan et al.^15^	Motivational interviewing or self‐management education	Unclear	Unclear	5 sessions and 6 sessions per week for motivational interviewing and self‐management education, respectively	Unclear	Group	Motivational interviewing: psychologist, diabetes nurse, nutrition expert, therapist. Self‐management education: unclear	N/R	Unclear
Cho et al.^29^	Internet‐based health gateway device	6 months	6 months	Recommendations based on participants self‐reported health data were sent every week for the first 3 months, then every other week for the last 3 months	Tailored recommendations offered to an individual	Individual	Nurse, diabetologist, dieticians, exercise experts, medical team	Various symptoms recorded as adverse events but none linked to the study intervention	Conventional outpatient management
Gerber et al.^20^	Mobile health	1 year	6,12,18,24 months	30–60 min at least every 2–3 months with the pharmacist and health coaches conducted monthly contact, home visits every other month, phone calls on alternating visits and messages between visits	Tailored to an individual	Individual	Pharmacists and health coaches	Assessed but none found	Usual diabetes care
Lake, 2020^27^2	Health promotion leaflet	Leaflet posted once	4 weeks	Leaflet mailed on a single occasion	Tailored for young adults living with T2D	Individual	N/R	Assessed but none found	Real‐world usual care
McElfish et al.^21^	Adapted family diabetes self‐management education	8 weeks	9 weeks, 6 and 12 months	Total of 10 h of culturally adapted education delivered in 75 min sessions	Culturally adapted to Marshallese adults	Individual + family members	Bilingual community health workers and certified diabetes educators	N/R	Standard diabetes self‐management education
Mukherji et al.^14^	Structured exercise	26 weeks	3 and 6 months	26 and 78 sessions for the once and thrice per week groups, respectively	Unclear	Group	Exercise training staff	N/R	Usual care
Middleton et al.^28^	Enhanced SMS‐based support and reminder programme	12 months	3,6,9,12 months	Two messages sent per week for the first 2 months, one message per week in the 3rd month then one text per month thereafter and a personalised appointment reminder	Tailored: Semi‐personalised messages based on baseline characteristics, gender and smoking status	Individual	Diabetes educator, endocrinologist, dietician	N/R	Clinic standard care
Essien et al.^19^	Intensive diabetes self‐management education	6 months	6 months	12 sessions, 2 h each, every 2 weeks	No tailoring of intervention was planned	Group	Doctors and nurses, also certified diabetes educators from endocrinology unit	N/R	Conventional education
Pyataket al.^26^	Occupational therapy intervention	6 months	6 months	Total of 10–16 h	Tailored to an individual	Individual + family member	Licensed occupational therapists, endocrinologists and licensed clinical social workers	11 serious adverse events recorded, none linked to the study intervention	Attention control group

Abbreviation: N/R, not reported.

### 3.2 Behaviour change techniques

The behaviour change techniques extracted from the studies were mapped and classified according to the BCT taxonomy (v1) of 93 hierarchically clustered techniques by Michie et al.^30^ Out of 16 domains, the majority of the studies used goals and planning (*n* = 8), shaping knowledge involving increasing knowledge and understanding on intended behaviour change and performance of a behaviour (*n* = 8), and feedback and monitoring (*n* = 7) among other BCTs in their interventions. Other BCTs utilised included social support (*n* = 4), regulation (*n* = 4), associations (*n* = 4), identity (*n* = 3), comparison of outcomes (*n* = 3), self‐belief (*n* = 2), comparison of behaviour (*n* = 2), natural consequences (*n* = 2) for example, providing information on the likely consequences of poor diabetes management to physical, emotional or overall health and well‐being and repetition and substitution (1) (Table S2).

Fewer BCTs were utilised in studies; the only studies included that reported statistically significant or clinically meaningful differences in clinical outcomes. In two studies, a combination of self‐monitoring of behaviour (feedback and monitoring) with shaping knowledge^19^ or goals and planning^14^ produced clinically meaningful but not statistically significant reductions in HbA1c of 22 mmol/mol (2.04%) and 12 mmol/mol (1.06%), respectively, between the intervention and control groups. Moreover, the aggregation of self‐monitoring of behaviour and goals and planning as active BCTs in one study^14^ resulted in a significant decrease in the waist circumference of young adults in the intervention group compared to the control group.

The theoretical frameworks underpinning the interventions, if any, were unclear in the majority of the studies (*n* = 9). One study^27^ utilised the intervention mapping framework for intervention development.

### 3.3 Risk of bias

Most of the randomised controlled trials (*n* = 6) were deemed to have some concerns, while the remaining two RCTs^14^, ^19^ were judged to be low risk. Domain 5; selection of reported results ranked poorly out of the five domains. Notably, two studies^20^, ^21^ were judged to be at high risk of this bias. Both quasi‐experimental studies^15^, ^22^ were categorised as having some concerns (moderate quality) due to bias related to confounding factors, intervention administration, participant retention and the assessment, detection and measurement of the outcomes.

### 3.4 Outcomes

#### 3.4.1 Primary outcome

##### Glycated haemoglobin (HbA1c)

Five studies^14^, ^15^, ^19^, ^20^, ^28^ (4 RCTs, 1 quasi‐experimental study) reported outcome data for HbA1c, with a total of 59 participants in the intervention groups and 62 participants in the control groups; the pooled mean difference (MD) between the groups was −1 mmol/mol (−0.1%); 95% CI: −8 mmol/mol (−0.7%) to 6 mmol/mol (0.5%) (Figure S1). The effect was not statistically significant (*p*‐value = 0.755). No heterogeneity was observed (*I* ^2^ = 0%; *p*‐value = 0.338). Subgroup analysis according to the quality of the studies (low risk vs. some concerns (*p*‐value; 0.751 and 0.902, respectively)) and mode of delivery (digital vs. non‐digital (*p*‐value; 0.900 and 0.750, respectively)) showed no statistically significant difference in the pooled mean difference between sub‐groups.

#### 3.4.2 Secondary outcomes

##### Other clinical outcomes

###### Body mass index (BMI)

Data for BMI were available for four RCT studies,^14^, ^20^, ^28^, ^29^ with 50 participants in the intervention groups and 59 in the control groups. The overall pooled MD was −0.248 kg/m^2^; 95% CI: −1.739 to 1.243. The effect was not significant (*p*‐value = 0.744); no between‐study heterogeneity was indicated by an *I* ^2^ of 0%; *p*‐value = 0.9506.

###### Blood pressure

Diastolic and systolic blood pressure events were reported in four RCT studies^14^, ^20^, ^21^, ^29^ with a combined total of 41 and 60 participants in the intervention and control groups, respectively. The pooled mean differences reported non‐significant decreases; pooled MD: −1.326 mmHg; 95% CI: −6.917 to 4.266; *p*‐value = 0.642 and pooled MD: −0.762 mmHg; 95% CI: −8.692 to 7.168; *p*‐value = 0.850, respectively. No heterogeneity was found in both cases; diastolic pressure: *I* ^2^ = 0%; *p*‐value = 0.901; systolic blood pressure: *I* ^2^ = 0%; *p*‐value = 0.994.

###### Weight

The pooled MD of two RCT studies^14^, ^29^ with a total of 21 and 27 participants in the intervention and control groups, respectively, reported a non‐significant decrease of −0.611 kilograms; 95% CI: −15.619 to 14.398; *p*‐value = 0.936. No heterogeneity was reported; *I* ^2^ = 0%; *p*‐value = 0.656.

###### Waist circumference

Similar to weight, the pooled MD of two RCT studies,^14^, ^29^ with a combined total of 21 and 27 participants in the intervention and control groups, respectively, reported a non‐significant decrease of −1.125 centimetres more in the intervention compared to control groups; 95% CI: −11.137 to 8.887; *p*‐value = 0.826. No heterogeneity was found, represented by an *I* ^2^ of 0%; *p*‐value = 0.330.

###### Body lipids

Triglycerides and total cholesterol outcome data were reported for 30 and 32 participants of the intervention and control groups in two RCT studies.^20^, ^28^ The pooled MD was not significant for both; total cholesterol‐pooled: MD; −2.815 mg/dL; 95% CI; −15.897 to 10.268; *p*‐value = 0.673; and triglycerides: pooled MD; −26.391 mg/dL; 95% CI; −60.586 to 7.803; *p*‐value = 0.130. No heterogeneity was observed for both; total cholesterol; *I* ^2^ = 0%; *p*‐value = 0.731; triglycerides: *I* ^2^ = 0%; *p*‐value = 0.859.

###### Fasting blood glucose

One study^15^ assessed fasting blood glucose (FBS), and despite a greater decrease in the FBS levels in the intervention group post‐intervention, compared to the control group, the results were not statistically significant (*p*‐value = 0.098).

###### Cholesterol

High‐ and low‐density lipoprotein were assessed in one study^20^; the levels did not statistically differ between the intervention and control groups; *p*‐value = 0.987 and *p*‐value = 0.322, respectively.

#### 3.4.3 Psychological health outcomes

##### Depression

The outcome data for depression were reported in two RCT studies^20^, ^27^ with a yielded total of 125 and 127 participants in the intervention and control groups. The standardised pooled MD showed a non‐significant decrease in depression among participants; pooled MD; −0.132; 95% CI; −0.378 to 0.114; *p*‐value 0.293. No heterogeneity was found; *I* ^2^ = 0%; *p*‐value 0.964.

##### Self‐efficacy

In three RCT studies,^20^, ^22^, ^28^ self‐efficacy outcome data were reported for 50 and 49 participants of the intervention and control groups. The pooled standardised MD; 0.427; 95% CI; −0.267 to 1.121 was not statistically significant (*p*‐value = 0.228). Moderate heterogeneity was observed; *I* ^2^ = 67.14%; *p*‐value = 0.054.

#### 3.4.4 Other psychological outcomes

No considerable differences were reported on diabetes distress in two studies, *p*‐value = 0.881,^20^; *p*‐value = 0.76,^28^; social support (*p*‐value = 0.632),^20^; quality of life (*p*‐value = 0.643),^20^; or stigma scores (*p*‐value = 0. 46)^28^ between the intervention and control groups.

#### 3.4.5 Behavioural outcomes

Studies that evaluated behavioural outcomes reported non‐significant differences in modifiable activity score (*p*‐value = 0.576),^14^ diabetes self‐care scores related to exercise (*p*‐value = 0.665),^20^ glucose testing (*p*‐value = 0.501)^20^; nevertheless, a trend towards enhanced dietary self‐care was observed (*p*‐value = 0.058).^20^ Contrasting findings were reported on the effect of SMIs on adherence to medication; in one study, significant improvements (*p* < 0.05) that were stable over 3 months were evidenced,^22^ while in another study,^20^ no significant differences (*p*‐value = 0.576) between the intervention and control groups were found. A tailored screening leaflet did not significantly improve the behavioural skills, perceived control and overcoming barriers in the intervention versus control groups (*p* > 0.05).^27^ In contrast, in another study, the overall clinic attendance in the intervention group was statistically different (log‐rank *p*‐value = 0.04) from the control group following personalised SMS text message reminders for each follow‐up appointment.^28^

##### Knowledge and attitude scores

Administration of a tailored leaflet promoting diabetes retinopathy screening did not produce significantly different results (*p* > 0.05) between the intervention and control groups in the knowledge scores regarding diabetes/vision link, retinal screening and attitude scores regarding retinal screening, risk perception, anticipated regret, normative beliefs and intention.^27^ However, knowledge of diabetes retinopathy was statistically different (*p* = 0.03) between the two groups.^27^

### 3.5 Publication bias

Publication bias was not statistically significant for HbA1c and BMI. Nevertheless, assessment of funnel plots indicated the presence of bias; studies with negative mean differences in HbA1c were scarcely reported, while small studies with positive mean differences and large studies with negative mean differences in BMI were scarcely reported. Evaluation of publication bias for weight, waist circumference, body lipids, depression and self‐efficacy was limited due to few studies.

## 4 DISCUSSION

In this review, self‐management interventions employed among young adults (18–45 years) living with Type 2 diabetes did not significantly improve clinical or psychological outcomes. Despite available recommendations for optimising behaviour change approaches for improvement in the health outcomes of young adults living with Type 2 diabetes,^31^ only 50% of the included studies were specifically targeted towards improving the health outcomes of this group. The findings in this review are consistent with those of a review by Wong et al., 2020, which reported no significant improvements in self‐care behaviours, clinical or psychological outcomes among young adults (15–39 years) with Type 1 and 2 diabetes.^8^ This review provides a more comprehensive evaluation of the effectiveness of SMIs on health outcomes of young adults with T2D, with 10 included studies as opposed to only two studies in Wong et al., 2020; thus, this review is the first to report these findings in this target group.

Contradicting results on the primary outcome, HbA1c, have been reported from other meta‐analyses that included the general adult population. In one review, patient‐centred self‐management interventions among adults 18–69 years resulted in a statistically significant decrease in HbA1c of 6 mmol/mol (0.56%) (95% CI; 9 to 4 mmol/mol [0.75% to 0.32%], *p* < 0.001).^32^ Similarly, another review of behavioural interventions reported a statistically significant improvement in HbA1c, post‐intervention of −3 mmol/mol (−0.31%) (95% C; −5 to −2 mmol/mol [−0.42 to −0.21%]), compared to usual care among adults <65 years.^33^ Further analysis of the interventions suggested that single‐component interventions may be more effective in younger adults (<65 years), following a clinically meaningful HbA1c reduction of −6 mmol/mol (−0.55%) (95% CI; −13 to 1 mmol/mol [−1.19% to 0.10%]).^33^ In contrast, multi‐component interventions appeared to benefit older adults (>65 years) more.^33^ The focus of these reviews on a specific type of self‐management intervention, patient‐centred or behavioural, may explain the contradicting findings to this review and imply that specific interventions may benefit younger adults. Meta‐regression to explore the effect of the type of intervention on outcomes in this review was limited due to few studies and heterogeneity in interventions used.

The pooled effect size of secondary outcomes in this meta‐analysis was not statistically significant. Notably, some of the included studies in the meta‐analysis cited a lack of power to detect changes in these outcomes.^21^, ^28^ Nevertheless, these findings are similar to the results reported by Captieux and Pierce, 2017, who found a non‐significant association between self‐management strategies and biomedical markers such as BMI, attributing it to the narrow focus on HbA1c as a primary outcome in most studies that may have resulted in the lack of reinforcement or less intensity of the self‐management strategies.^10^ Furthermore, the findings of secondary outcomes were imprecise due to fewer reporting studies and considerably small sample sizes of the young adults.

Subgroup analysis found no significant subgroup effect between interventions delivered digitally or non‐digitally. However, this finding should be interpreted with caution due to low power attributed to the small number of studies (*n* = 5) included in the analysis. Adequate statistical power for subgroup analysis in meta‐analysis, according to power calculations, would require dozens to hundreds of studies.^34^ Digital interventions have been suggested for young adults with T2D, especially for their mental health, as they can provide readily available and accessible support services that are limited in traditional face‐to‐face services.^25^ Furthermore, these interventions are effective in reducing psychological support barriers such as accessibility and stigma,^35^ which are evidenced barriers to self‐management programme attendance^36^ and adherence^37^ among young people living with T2D. Despite the advantages offered by digital interventions, careful consideration is needed to prevent inequities due to exclusion, including those related to digital literacy, accessibility or engagement,^38^ which can increase the digital divide among the targeted group.

The importance of tailoring interventions has been highlighted for populations such as young adults living with T2D. In their review, Wong et al., 2020, recommended that future interventions targeting young adults with diabetes should consider their preferences, concerns and expectations, while also addressing the unique challenges they face.^8^ Our review found that half of the interventions utilised elements of tailoring for the target group, including specifically designing the intervention for young adults with T2D, which is one of three ways of tailoring interventions for this group.^31^ The remaining interventions were tailored based on one or more individual characteristics, goals, preferences or needs; a more individual‐centred approach termed individualised care,^39^ although some authors describe this approach as tailored intervention.^40^ In our review, at study level, both tailored and non‐tailored interventions statistically improved some health outcomes among the young adults, suggesting no differences in this regard. However, the studies that employed tailored interventions reported key methodological limitations, including low sample sizes, low recruitment rates, moderate to high baseline scores and a lack of statistical power to detect changes in outcomes, which may have affected the reported effectiveness of their interventions. More high‐quality, tailored interventions are needed to determine their effectiveness among young adults living with T2D.

### 4.1 Behaviour change techniques

Various behaviour change techniques (BCTs), ranging from two to nine, were utilised in the included studies, aligning with existing self‐management reviews.^8^, ^41^ The main domains were goals and planning, shaping knowledge and feedback and monitoring, which correspond with other reviews.^8^, ^42^ Feedback and monitoring, particularly self‐monitoring of behaviour, were the common BCTs among studies that reported clinically meaningful reductions in HbA1c.^14^, ^19^ A meta‐regression of BCTs in behavioural interventions linked self‐monitoring of behaviour to improved intervention effectiveness.^43^ Effectiveness was further improved if self‐monitoring of behaviour was combined with one or more self‐regulating techniques such as goal setting, reviewing behaviour goals, forming intentions and providing feedback on behaviour.^43^ Actively tracking health outcomes by individuals living with T2D improves self‐regulation, emotional investment and control of the condition, which are critical for self‐management.^44^

In our review, behavioural interventions utilising the fewest (*n* = 2) combined BCTs led to clinically meaningful reductions in HbA1c levels and statistically significant decreases in waist circumference among the target group.^14^, ^19^ Conversely, other reviews of behavioural studies have shown that a greater number of BCTs are associated with more significant improvements in blood glucose control^45^ and weight loss,^46^ particularly in adults aged 18 and those between 40 and 69 years. Michie et al. suggested that a higher number of techniques may negatively affect the quality and fidelity of interventions,^43^ which in turn may affect their overall effectiveness. Therefore, using fewer BCTs in self‐management interventions may be more beneficial for young adults with T2D. Nevertheless, minimal evidence exists on the most effective aggregation of BCTs for self‐management interventions among this group; more research on BCTs using a reliable taxonomy is needed.

Despite evidence suggesting that interventions based on a theoretical background or framework are more effective in achieving lasting glycaemic control among adults compared to those without it,^47^, ^48^ the theoretical underpinning of the interventions in our review was only done in one study. The use of behaviour change theory or frameworks is critical for the improvement of the design of interventions and understanding of behaviour change mechanisms that are effective.^49^

### 4.2 Strengths and limitations

This review boasts in its extensive search strategy, precise and reliable review methods and selection of studies, quality assessment and data extraction that involved two or more independent reviewers. Furthermore, to the best of our knowledge, this is the first systematic review that evaluated the effectiveness of self‐management interventions among young adults with T2D and behaviour change techniques linked to improved outcomes. This review has some limitations. At the study level, the reporting of BCTs was poorly done. Consequently, some BCTs may have been missed in the synthesis process. Moreover, outcome data were characterised by much missing data, and some studies were not powered enough to detect changes in secondary outcomes. Lastly, limited studies, four out of nine of those included in the meta‐analysis, targeted only young adults living with T2D. Therefore, findings from this review are imprecise and inconclusive on the effectiveness of self‐management interventions among young adults under 45 years living with T2D.

## 5 CONCLUSION

Our review highlights that current self‐management interventions did not improve clinical and psychological outcomes among young adults (18–45 years) living with T2D. More effective strategies are necessary, specifically targeted at this priority population.

## AUTHOR CONTRIBUTIONS

This study was conceived by MH and CG; the scope and search strategy were designed by SK, MH and CG. Database search was conducted by SK, and SK, JM, JH, SSO, SH, ZK, SN and CG screened the titles, abstracts and full texts of eligible articles. Quality assessment was done by SK and CG, and data extraction was done by SK and JM. SK and CG conducted the statistical analysis. The first draft of the article was prepared by SK under the supervision of MH and CG.

## FUNDING INFORMATION

This work was supported by the University of Leicester through the Future 50 as part of a PhD studentship. The funder of the project had no role in the study design, data collection, data analysis, data interpretation or writing of the report.

## CONFLICT OF INTEREST STATEMENT

None declared.

## ETHICS STATEMENT

Not applicable. Data utilised are in the public domain.

## Supporting information


Data S1:

## Data Availability

Publicly available data were involved in the study.
