# Supplementary material for: Effectiveness of self‐management interventions on Type 2 diabetes among young adults (18–45 years): A systematic review and meta‐analysis
Source: Diabet Med. 2025 Aug 21;42(10):e70127. doi: 10.1111/dme.70127 (PMC12434426; doi:10.1111/dme.70127)
Supplement: Supplementary file 1 — Data S1: [file DME-42-e70127-s001.docx]

**Table S1: Search strategies**

**MEDLINE via Ovid**

| 1 | (diabetes adj3 type 2).ti. |
| --- | --- |
| 2 | (diabetes adj3 type two).ti. |
| 3 | (diabetes adj3 type ii).ti. |
| 4 | exp Diabetes Mellitus, Type 2/ |
| 5 | non-insulin-dependent diabetes mellitus.ti. |
| 6 | stable diabetes mellitus.ti. |
| 7 | adult-onset Diabetes Mellitus.ti. |
| 8 | (NIDDM or T2DM or T2D).ti. |
| 9 | (glyc?mic adj3 control).ti. |
| 10 | 1 or 2 or 3 or 4 or 5 or 6 or 7 or 8 or 9 |
| 11 | exp Self Care/ or exp Self-Management/ |
| 12 | self management.mp. |
| 13 | self care.mp. |
| 14 | 11 or 12 or 13 |
| 15 | exp randomized controlled trial/ |
| 16 | controlled clinical trial.pt. |
| 17 | randomized.ab. |
| 18 | placebo.ab. |
| 19 | clinical trials as topic/ |
| 20 | randomly.ab. |
| 21 | trial.ti. |
| 22 | 15 or 16 or 17 or 18 or 19 or 20 or 21 |
| 23 | 10 and 14 and 22 |
| 24 | gestational diabetes.mp. or exp Diabetes, Gestational/ |
| 25 | prediabetes.mp. or exp Prediabetic State/ |
| 26 | 24 or 25 |
| 27 | 23 not 26 |
| 28 | limit 27 to english language |

**Cochrane Library**

| #1 | (diabetes NEAR/3 ("type 2" or "type ii" or "type two")):ti |
| --- | --- |
| #2 | MeSH descriptor: [Diabetes Mellitus, Type 2] explode all trees |
| #3 | ("non-insulin-dependent diabetes mellitus"):ti |
| #4 | ("stable diabetes mellitus"):ti |
| #5 | ("adult-onset Diabetes Mellitus"):ti |
| #6 | (NIDDM or T2DM or T2D):ti |
| #7 | (("glycemic control")):ti |
| #8 | #1 or #2 or #3 or #4 or #5 or #6 or #7 |
| #9 | MeSH descriptor: [Self-Management] explode all trees |
| #10 | MeSH descriptor: [Self Care] explode all trees |
| #11 | ("self management"):ti,ab,kw |
| #12 | ("self care"):ti,ab,kw |
| #13 | #9 or #10 or #11 or #12 |
| #14 | #8 and #13 |
| #15 | MeSH descriptor: [Diabetes, Gestational] explode all trees |
| #16 | MeSH descriptor: [Prediabetic State] explode all trees |
| #17 | ("gestational diabetes mellitus"):ti,ab,kw |
| #18 | (prediabetes):ti,ab,kw |
| #19 | #15 or #16 or #17 or #18 |
| #20 | #14 not #19 |
| #21 | Limit to Trials |
| #22 | Limit to English |

**CINAHL**

| S19 | Limit to English |
| --- | --- |
| S18 | S14 NOT S17 |
| S17 | S15 OR S16 |
| S16 | (MH "Prediabetic State") OR "prediabetes" |
| S15 | (MM "Diabetes Mellitus, Gestational") OR "gestational diabetes" |
| S14 | S8 AND S12 AND S13 |
| S13 | ((MH "Experimental Studies+") OR (MH "Multicenter Studies") OR (MH "Random Sample+") OR (MH "Placebos") OR (MH "Control (Research)+") OR (MH "Crossover Design") OR ((TI random* OR AB random*) OR (TI sham OR AB sham) OR (TI placebo* OR AB placebo*)) OR (((TI singl* OR AB singl*) OR (TI doubl* OR AB doubl*)) W1 ((TI blind* OR AB blind*) OR (TI dumm* OR AB dumm*) OR (TI mask* OR AB mask*))) OR (((TI tripl* OR AB tripl*) OR (TI trebl* OR AB trebl*)) W1 ((TI blind* OR AB blind*) OR (TI dumm* OR AB dumm*) OR (TI mask* OR AB mask*))) OR ((TI control* OR AB control*) N3 ((TI study OR AB study) OR (TI studies OR AB studies) OR (TI trial* OR AB trial*) OR (TI group* OR AB group*))) OR ((TI clinical OR AB clinical) N3 ((TI study OR AB study) OR (TI studies OR AB studies) OR (TI trial* OR AB trial*))) OR ((TI Nonrandom* OR AB Nonrandom*) OR (TI "non random*" OR AB "non random*") OR (TI "non-random*" OR AB "non-random*") OR (TI "quasi-random*" OR AB "quasi-random*") OR (TI quasirandom* OR AB quasirandom*)) OR ((TI phase OR AB phase) N6 ((TI study OR AB study) OR (TI studies OR AB studies) OR (TI trial* OR AB trial*))) OR (((TI crossover OR AB crossover) OR (TI "cross-over" OR AB "cross-over")) N3 ((TI study OR AB study) OR (TI studies OR AB studies) OR (TI trial* OR AB trial*))) OR (((TI multicent* OR AB multicent*) OR (TI "multi-cent*" OR AB "multi-cent*")) N3 ((TI study OR AB study) OR (TI studies OR AB studies) OR (TI trial* OR AB trial*))) OR (TI allocated OR AB allocated) OR (((TI "open label" OR AB "open label") OR (TI "open-label" OR AB "open-label")) N5 ((TI study OR AB study) OR (TI studies OR AB studies) OR (TI trial* OR AB trial*))) OR (((TI equivalence OR AB equivalence) OR (TI superiority OR AB superiority) OR (TI "non-inferiority" OR AB "non-inferiority") OR (TI noninferiority OR AB noninferiority)) N3 ((TI study OR AB study) OR (TI studies OR AB studies) OR (TI trial* OR AB trial*))) OR ((TI "pragmatic study" OR AB "pragmatic study") OR (TI "pragmatic studies" OR AB "pragmatic studies")) OR (((TI pragmatic OR AB pragmatic) OR (TI practical OR AB practical)) N3 (TI trial* OR AB trial*)) OR (((TI quasiexperimental OR AB quasiexperimental) OR (TI "quasi-experimental" OR AB "quasi-experimental")) N3 ((TI study OR AB study) OR (TI studies OR AB studies) OR (TI trial* OR AB trial*))) OR (TI trial)) |
| S12 | S9 OR S10 OR S11 |
| S11 | self care |
| S10 | self management |
| S9 | (MH "Self-Management") OR (MH "Self Care") |
| S8 | S1 OR S2 OR S3 OR S4 OR S5 OR S6 OR S7 |
| S7 | TI (NIDDM or T2DM or T2D) |
| S6 | TI adult-onset diabetes mellitus |
| S5 | TI stable diabetes mellitus |
| S4 | TI non-insulin dependent diabetes mellitus |
| S3 | TI (diabetes N3 (type 2 or type ii or type two)) |
| S2 | (MM "Glycemic Control") |
| S1 | (MH "Diabetes Mellitus, Type 2") |

**Web of Science**

| 1 | (((((TI=(diabetes NEAR/3 type 2 or type ii or type two)) OR TI=(type 2 diabetes mellitus)) OR TI=(non-insulin-dependent diabetes mellitus)) OR TI=(stable diabetes mellitus)) OR  TI=(adult-onset diabetes mellitus)) OR TI=(NIDDM or T2DM or T2D) OR TI=(glyc?mic  control) |
| --- | --- |
| 2 | ((TS=(self management )) OR TS=(self care )) |
| 3 | TS=(randomised OR randomized OR randomisation OR randomization OR placebo* OR (random* AND (allocat* OR assign*) ) OR (blind* AND (single OR double OR treble OR triple) )) |
| 4 | #3 AND #2 AND #1 |
| 5 | (TS=(gestational diabetes )) OR TS=(prediabetes) |
| 6 | #4 NOT #5 |
| 7 | Limit to English |

**SCOPUS**

| 1 | (TITLE (diabetes W/3 ( "type 2" OR "type ii" OR "type two" )) OR TITLE ("type 2 diabetes mellitus" OR "non-insulin dependent diabetes mellitus" OR "stable diabetes mellitus" OR "adult-onset diabetes mellitus" OR niddm OR t2dm OR t2d OR "glyc?mic control")) |
| --- | --- |
| 2 | TITLE-ABS-KEY ( {self care} OR {self management} OR {self-care} OR {self-management}) |
| 3 | TITLE-ABS-KEY ( random* OR sham OR placebo* ) OR TITLE-ABS-KEY ( ( singl* OR doubl* ) W/1 ( blind* OR dumm* OR mask* ) ) OR TITLE-ABS-KEY ( ( tripl* OR trebl* ) W/1 ( blind* OR dumm* OR mask* ) ) OR TITLE-ABS-KEY ( control* W/3 ( study OR studies OR trial* OR group* ) ) OR TITLE-ABS-KEY ( clinical W/3 ( study OR studies OR trial* ) ) OR TITLE-ABS-KEY ( nonrandom* OR "non random*" OR non-random* OR quasi-random* OR quasirandom* ) OR TITLE-ABS-KEY ( phase W/6 ( study OR studies OR trial* ) ) OR TITLE-ABS-KEY ( ( crossover OR cross-over ) W/3 ( study OR studies OR trial* ) ) OR TITLE-ABS-KEY ( ( multicent* OR multi-cent* ) W/3 ( study OR studies OR trial* ) ) OR TITLE-ABS ( allocated ) OR TITLE-ABS-KEY ( ( "open label" OR open-label ) W/5 ( study OR studies OR trial* ) ) OR TITLE-ABS-KEY ( ( equivalence OR superiority OR non-inferiority OR noninferiority ) W/3 ( study OR studies OR trial* ) ) OR TITLE-ABS-KEY ( "pragmatic study" OR "pragmatic studies" ) OR TITLE-ABS-KEY ( ( pragmatic OR practical ) W/3 trial* ) OR TITLE-ABS-KEY ( ( quasiexperimental OR quasi-experimental ) W/3 ( study OR studies OR trial* ) ) OR TITLE ( trial ) OR KEY ( trial ) |
| 4 | 1 AND 2 AND 3 |
| 5 | TITLE-ABS-KEY(gestational diabetes OR prediabetes) |
| 6 | 4 AND NOT 5 |
| 7 | Limit to English |

**Table S2: Behaviour change techniques utilised in the self-management interventions**

| Behaviour Change Technique | Abdollahi (2020)^22^ | Dibaiyan  (2022)^15^ | Cho (2017)^29^ | Gerber (2023)^20^ | Lake (2020)^27^ | McElfish (2021)^21^ | Mukherji (2022)^14^ | Middleton (2021)^28^ | Essien (2017)^19^ | Pyatak (2018)^26^ |
| --- | --- | --- | --- | --- | --- | --- | --- | --- | --- | --- |
| Goals & Planning | ✓ | ✓ |  | ✓ | ✓ | ✓ | ✓ | ✓ |  | ✓ |
| Feedback & Monitoring | ✓ | ✓ | ✓ | ✓ |  |  | ✓ | ✓ | ✓ |  |
| Social Support |  |  |  | ✓ |  | ✓ |  | ✓ |  | ✓ |
| Shaping Knowledge | ✓ |  | ✓ | ✓ | ✓ | ✓ |  | ✓ | ✓ | ✓ |
| Natural Consequences | ✓ |  |  |  | ✓ |  |  |  |  |  |
| Comparison of Behaviour |  |  |  |  | ✓ | ✓ |  |  |  |  |
| Associations |  |  | ✓ | ✓ |  |  |  | ✓ |  | ✓ |
| Repetition & Substitution |  |  | ✓ |  |  |  |  |  |  |  |
| Comparison of Outcomes | ✓ | ✓ |  |  | ✓ |  |  |  |  |  |
| Reward & Threat |  |  |  |  |  |  |  |  |  |  |
| Regulation | ✓ | ✓ |  |  |  |  |  | ✓ |  | ✓ |
| Antecedents |  |  |  |  |  |  |  |  |  |  |
| Identity | ✓ | ✓ |  |  | ✓ |  |  |  |  |  |
| Scheduled Consequences |  |  |  |  |  |  |  |  |  |  |
| Self-belief |  |  |  | ✓ | ✓ |  |  |  |  |  |
| Covert Learning |  |  |  |  |  |  |  |  |  |  |

**Figure S1: Forest plot of estimated effect sizes of health outcomes**

**
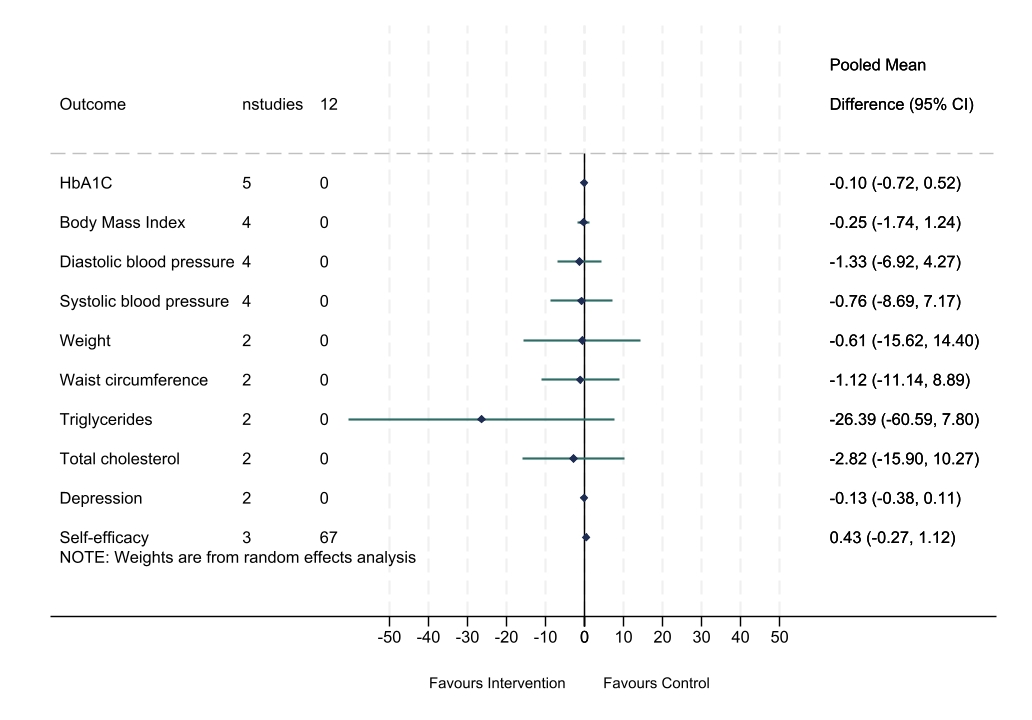
**
